# Managing High-Acuity Risk Through MDT Learning and Proactive Practice: Incident Trends on a Female PICU Over 12 Months (January–December 2025)

**DOI:** 10.1192/bjo.2026.11623

**Published:** 2026-06-30

**Authors:** Jason Tan, Sujata Sharma, Adele Lee Malik, Jennifer Beal, Omer Malik

**Affiliations:** Cygnet Healthcare, London, United Kingdom

## Abstract

**Aims:**

**Background:** Female Psychiatric Intensive Care Units (PICUs) manage patients with high levels of acuity, behavioural disturbance and complex risk. In this setting, safety is supported not only through containment but through proactive MDT practice, reflective learning and patient-centred approaches.

**Aim:** To analyse incident trends over a 12-month period on a female PICU (Svanna) at Cygnet Beckton and to describe MDT practices supporting risk management and learning in a high-acuity environment.

**Methods:**

A retrospective descriptive analysis of Datix-reported incidents was undertaken for January–December 2025. Monthly data were reviewed across violence and aggression, self-harm, security incidents (including AWOL and attempted AWOL), medication-related incidents, and injury or clinical deterioration events. MDT learning and practice developments were collated through service review.

**Results:**

Svanna ward recorded 1108 incidents during 2025 (monthly range 50–144).

Violence and aggression accounted for 571 incidents, including 386 episodes of actual physical violence. Physical violence was highest in January (56 incidents) and reduced during mid-year to 13 incidents in August, before increasing again towards year end (29 incidents in December). The fluctuation in violence and aggression is due to the complexity of referrals with comorbid neuro divergent background.

Self-harm incidents totalled 382, predominantly head banging (233) and swallowing (30), peaking in September (67 incidents).

Security incidents totalled 58, including attempted AWOL/abscond (11) and AWOL (3). Medication-related incidents were 26, while injury and clinical deterioration incidents totalled 30.

**Conclusion:**

**MDT good practice and learning:**

In response to high acuity, Svanna adopted proactive MDT-led approaches including frequent reviews outside scheduled ward rounds, DBT-informed clinical skills within the medical team, close nursing–medical collaboration, and patient involvement in medication planning where clinically appropriate.

Following serious or repeated incidents, After Action Reviews were used to support shared understanding of risk, staff reflection and identification of system-level learning. Lessons learned informed changes to observation levels, engagement strategies, handover quality and MDT communication, supporting adaptive risk management in a PICU context. Further to that, the ward has been implementing sensory-informed approaches which include environmental adaptations, structured sensory assessment and workforce development initiatives which has been helpful to promote emotional regulation, reduce incidents and enhance therapeutic engagement.

**Conclusion:**

Despite high baseline acuity, this review demonstrates periods of reduced violence and stabilisation within a female PICU, supported by reflective MDT practice and structured learning following incidents.

